# Formyl peptide receptor 2 orchestrates mucosal protection against *Citrobacter rodentium* infection

**DOI:** 10.1080/21505594.2019.1635417

**Published:** 2019-07-04

**Authors:** S. Sharba, V. Venkatakrishnan, M. Padra, M. Winther, M. Gabl, M. Sundqvist, J. Wang, H. Forsman, S. K. Linden

**Affiliations:** a Department of Medical Biochemistry and Cell Biology, Sahlgrenska Academy, Gothenburg, Sweden; b Department of Rheumatology and Inflammation Research, Sahlgrenska Academy, Gothenburg, Sweden; c Cancer and Inflammation Program, National Cancer Institute at Frederick, Frederick, MD, USA

**Keywords:** Mucin, mucus, Citrobacter rodentium, enteropathogenic *Escherichia coli*, Fpr2, colitis

## Abstract

*Citrobacter rodentium* is an attaching and effacing intestinal murine pathogen which shares similar virulence strategies with the human pathogens enteropathogenic- and enterohemorrhagic *Escherichia coli* to infect their host. *C. rodentium* is spontaneously cleared by healthy wild-type (WT) mice whereas mice lacking Muc2 or specific immune regulatory genes demonstrate an impaired ability to combat the pathogen. Here we demonstrate that apical formyl peptide receptor 2 (Fpr2) expression increases in colonic epithelial cells during *C. rodentium* infection. Using a conventional inoculum dose of *C. rodentium*, both WT and Fpr2^−/−^ mice were infected and displayed similar signs of disease, although Fpr2^−/−^ mice recovered more slowly than WT mice. However, Fpr2^−/−^ mice exhibited increased susceptibility to *C. rodentium* colonization in response to low dose infection: 100% of the Fpr2^−/−^ and 30% of the WT mice became colonized and Fpr2^−/−^ mice developed more severe colitis and more *C. rodentium* were in contact with the colonic epithelial cells. In line with the larger amount of *C. rodentium* detected in the spleen in Fpr2^−/−^ mice, more *C. rodentium* and enteropathogenic *Escherichia coli* translocated across an *in vitro* mucosal surface to the basolateral compartment following FPR2 inhibitor treatment. Fpr2^−/−^ mice also lacked the striated inner mucus layer that was present in WT mice. Fpr2^−/−^ mice had decreased mucus production and different mucin *O-*glycosylation in the colon compared to WT mice, which may contribute to their defect inner mucus layer. Thus, Fpr2 contributes to protection against infection and influence mucus production, secretion and organization.

## Introduction

The mucosal surface of the gastrointestinal (GI) tract is constantly exposed to pathogens and antigens from food and water, and the mucus layer covering the epithelial cells forms a barrier that separates luminal content from the mucosa to protect against such insult. The mucus layer contains an array of defensive compounds, is constitutively produced by goblet cells and dynamically regulated by both the innate and adaptive immune system during infection [1]. The mucus layer is divided into an outer non-sterile and an inner nominally sterile layer covering the mucosa [2]. Knockout studies of specific mucins in mice have shown an increased susceptibility to bacterial infections along the GI tract [3–5]. Nevertheless, GI pathogens have evolved a multitude of strategies to circumvent the protective response by the host [6].

Attaching and effacing (A/E) bacteria, enteropathogenic *Escherichia coli* (EPEC) and enterohaemorrhagic *E. coli* (EHEC) cause acute watery diarrhea and hemorrhagic colitis, respectively, in humans [7]. Due to their poor infectivity in mice, a related mouse pathogen, *Citrobacter rodentium* (*C. rodentium*) is commonly used to study the pathogenesis of A/E bacteria *in vivo* [8]. This group of pathogens shares a common strategy where infection of the host is initiated by colonization and attachment to the intestinal epithelium followed by effacement of the microvilli and establishment of actin-rich pedestal-like structures [8,9]. A type 3 secretion system is required to successfully translocate effector proteins into host epithelial cells to manipulate the host actin cytoskeleton thereby establishing the characteristic pedestal-like structures [9–11]. Collectively, these events likely account for coexisting epithelial hyperplasia and diarrhea due to the manipulation of host epithelial cells and interference with normal cellular functions of the distal colon and rectum [12].

Chemoattractants, including the N-formyl peptides derived from the host or the invading pathogen, govern recruitment of phagocytes to sites of inflammation and infection [13,14]. The formyl peptides bind to formyl peptide receptors (FPRs), belonging to the class of heterotrimeric G-protein-coupled receptors (GPCRs) present on the surface of activated phagocytes [15]. In humans/mice, FPR1/Fpr1 and FPR2/Fpr2 have received much attention for their roles in host defense against bacterial infections [16–20]. Apart from being expressed by phagocytes and regulating their functions, FPRs have been found to be expressed by both human and mouse intestinal epithelial cells [17,21]. Fpr2 expressed on mouse colonic epithelial cells becomes upregulated following dextran-sulfate sodium (DSS) treatment and mice lacking Fpr2 (Fpr2^−/−^) show increased susceptibility to DSS and fail to recover from the ensuing insult [17]. Human GI epithelial cells also express FPR2, and formylated bacterial peptides have been shown to accelerate healing in mechanically injured monolayers from such cell types *in vitro* [22,23]. Cytokines, such as interleukin (IL)-4, IL-6, IL-13, and interferon-γ (IFN-γ) have been shown to enhance FPR2 gene expression of *in vitro* cultured human intestinal epithelial cells [24]. Taken together, these studies indicate that FPR2/Fpr2 play important roles in protecting the host against GI diseases. However, the effect of Fpr2 on the mucus barrier and susceptibility to *C. rodentium* infection in the presence or absence of the receptor have not yet been examined.

The aims of this study were to investigate the localization of Fpr2 following *C. rodentium* infection in WT mice and to examine whether the absence of Fpr2 increases the susceptibility to *C. rodentium* infection. We analyzed a number of aspects important for infection and the development of colitis, including morphological alterations of epithelial cells in the distal colon, mucus production and secretion, pathogen density, and bacterial dissemination. Our data demonstrate that Fpr2^−/−^ mice are more susceptible to *C. rodentium* infection with an increased number of pathogens in contact with epithelial cells and translocation to the periphery during the early phase of infection. Furthermore, we verified a role for human epithelial FPR2 in limiting bacterial translocation using *in vitro* colonic mucosal surfaces.

## Methods

### Animals

Eight- to 12-week-old male C57BL/6 mice were purchased from Charles Rivers (Germany) and Formyl Peptide Receptor 2 Knock-out (Fpr2^−/−^) mice were generated as described previously [25]. They were housed under pathogen-free conditions at the Department of Rheumatology and Inflammation Research, University of Gothenburg, Sweden. The animals had free access to water and food throughout the experiment and were monitored daily for the duration of the study. Experimental procedures were approved by the Swedish Laboratory Animal Ethical Committee based on the regulation of the Swedish National Board for Laboratory Animals.

### Infection of mice with *C. rodentium*

*C. rodentium* strain ICC169 was cultured in Luria-Bertani (LB) broth (CM0996, Oxoid) over-night (O/N) at 37°C and mice were inoculated with 0.2 mL of the bacterial suspension containing 5 × 10^9^ colony forming units (CFU)/mouse for the conventional dose and 5 × 10^7^ CFU/mouse for low dose by oral gavage. Mice were weighed and CFUs were quantified by counting *C. rodentium* ~1 mm diameter fuschia-colored colonies cultured on MacConkey agar (CM0007, Oxoid) O/N to confirm the CFU inoculum. Fecal homogenates were plated and grown for 20 h at 37°C. Control and infected mice were anesthetized with isoflurane and sacrificed by CO_2_ and cervical dislocation at day 6 (for low dose), 10, 14, and 19 post infection (PI). The most distal 6 cm of the distal colon was harvested. Thereof, the most distal 1 cm specimen starting at the anal verge was collected and immediately stored at −80°C for glycosylation analysis, and the next 1 cm was collected in Carnoy´s methanol fixative (60% dry methanol, 30% chloroform, and 10% glacial acetic acid). The following 1.5 cm section was collected in 1 mL RNA*later* (AM7020, Thermo Fisher Scientific) and the final 1.5 cm section in the perfusion-extraction buffer for cytokine analysis (specified in cytokine protein section, below).

### Cell culture, *C. rodentium* infection, and FPR2 inhibitor treatment

HT29 MTX-E12 cells were maintained and grown into an *in vitro* mucosal surface as described [26,27]. Briefly, cells were grown to confluency on snap-well plates (1.12 cm^2^, CLS3407, Corning®), subjected to differentiating media for 6 d and then for semi-wet interface culture with mechanical stimulation for 28 d. For FPR2 inhibitor and infection with EPEC and *C. rodentium*, cell culture media were changed to antibiotic-free RPMI 1640 (BE12-702F/U1 Lonza) + 10% Fetal bovine serum (FBS) (34–015-CV, Corning®) at day 34 post-confluency. The extended time of culture was to ensure homogeneity, improve morphology and allow the cells to produce a mucus layer prior to infection. *C. rodentium* was grown as described above and harvested into sterile PBS. For infection, 10 µL of *C. rodentium* and EPEC suspensions with a CFU count of 2 × 10^9^ and 4 × 10^6^ CFU/mL, respectively, were added to the apical side of the membrane 24 h prior to harvest in Carnoy´s methanol fixative. For inhibiting FPR2, 1 µM of HF965A (ref PMID: 25462815) kindly provided by Henrik Franzyk (Department of Drug Design and Pharmacology, University of Copenhagen, Denmark), PBP10 (Caslo Laboratory) or WRW4 (GenScript Corp) was added to the basolateral compartment 2 h prior to infection. The inhibitors were replenished at 2 hPI to avoid complete degradation of the peptide during the experiment.

### Histology

For analysis, 5-µm-thick paraffin-embedded mouse distal colon and HT29 MTX-E12 *in vitro* mucosal membrane sections were warmed for 10 min at 60°C, deparaffinized and rehydrated in xylene for 30 min and subsequently in decreasing concentrations of ethanol for 2 min each; 100%, 95%, 70% and distilled H_2_O and stained with Hematoxylin/eosin (H&E) for analysis. The slides were blinded and the entire specimens were systematically scored for crypt architecture (0–4), tissue damage (0–4), crypt length (0–4), neutrophil count in lamina propria (0–4), inflammatory cell infiltration (0–4), and crypt abscesses (0–4). Goblet cell depletion and mucus-related parameters were verified by Periodic-acid Schiff (PAS) (3952016, Sigma-Aldrich)/Alcian blue 8GX (A5268, Sigma-Aldrich) stain as described in [26]. The total colitis scores reflect the sum of these scores.

### Immunofluorescence

Paraffin-embedded sections were deparaffinized and rehydrated as described above. For antigen retrieval, sections were heated in 0.01 M citric acid buffer pH 6 at 99°C for 30 min and 10 min for tissues and *in vitro* mucosal membranes, respectively, and allowed to cool down for 30 min at room temperature (RT). Non-specific binding was blocked using 5% FBS diluted in phosphate-buffered saline (PBS) and the sections were incubated with an antibody recognizing the murine and human MUC2 mucin (polyclonal rabbit-anti-MUC2C3 [2], kind gift from Professor G. Hansson, University of Gothenburg, Sweden) diluted (1:1000) in blocking buffer and incubated for 2 h, at RT. For bacterial localization, sections were incubated with monoclonal rabbit-anti-*E. coli* O152 antiserum (295,774, Denka Seiken) diluted 1:100 O/N at 4°C. For Fpr2 localization, sections were incubated with the primary antibody rabbit-anti-FPRL1 (NLS1878, Novus Biologicals) diluted 1:800 in protein block (X0909, DAKO) and incubated at 4°C O/N. Sections were rinsed three times in PBS and incubated for 1 h, at RT with Alexa Fluor 488-conjugated goat anti-rabbit (1:500) followed by three washes in PBS. In situ hybridization of eubacteria in tissues and *C. rodentium* on *in vitro* mucosal membranes was carried out as described in [26] using a Cy3.5 5ʹ labeled eubacteria-specific probe. To outline the tissue sections, Cell mask (C10046, Thermo Fisher Scientific) diluted (1:14,000) in PBS was applied and sections were incubated for 30 min at RT followed by a brief wash in PBS and distilled H_2_O. To visualize DNA, the sections were mounted with DAPI containing Prolong Gold anti-fade reagent (P36935, Thermo Fisher Scientific). Pictures were captured with an Eclipse 90i fluorescence microscope (Nikon). Semi-quantification of bacteria in the different locations of the tissue sections was scored based on Eub338 density in the inner mucus layer/in close association with epithelial cells (Score 1 = >10%, 2 = 10–50%, 3 = 50–70% and 4 = >70%) and the total number of crypts colonized below the neck of the crypt (Score 1 = 1–3 crypts, 2 = 3–5 crypts and 3 = >5 crypts colonized). Semi-quantification of Fpr2 at the surface epithelium in WT mice was scored based on percentage of epithelium stained by the Fpr2 antibody in 10 fields of view (1 = >20%, 2 = 20–40%, 3 = 40–60%, 4 = 60–80% and 5 = >80%). Staining was scored in a blinded fashion at 40x magnification.

### Incorporation of GalNAz

A total volume of 0.5 mL per mouse of the Click-iT reagent N-azidoacetylgalactosamine (GalNAz) (C33365, Thermo Fischer Scientific) was prepared by dissolving 2.6 mg of GalNAz in 100 µL Dimethyl sulfoxide (A3672, AppliChem GmbH) and diluted in 1 mL in PBS (0.15 mol/L NaCl, 5 mmol/L sodium phosphate buffer, pH 7.4). The injections were given intraperitoneally. Mice were sacrificed 3 h after the GalNAz injection. Paraffin-embedded samples were dewaxed, hydrated, and washed in PBS. Membrane sections were incubated with 20 µL of the reaction mix from the tetramethylrhodamine (TAMRA) glycoprotein detection kit (C10410, Thermo Fischer Scientific) and incubated at room temperature for 2 h, followed by one wash in PBS. The sections were mounted with DAPI containing Prolong Gold anti-fade reagent (P36935, Thermo Fisher Scientific) to visualize DNA (nuclei). The intensity and localization of freshly produced mucins were evaluated in a blinded fashion at 40x magnification on an Eclipse 90i fluorescence microscope (Nikon). Due to that co-stain with antibody against Muc2 affected the TAMRA intensity; goblet cells were identified based on cellular morphology.

### Cytokine protein detection

Intestinal colon samples were collected as described above in inhibition buffer containing; Soybean Trypsin Inhibitor (T9003, Sigma Aldrich), Pefabloc (399–01, Coatech), EDTA, BSA (A-4503, Sigma Aldrich), and PBS-tween and stored at −80°C. For protein detection of cytokines and chemokines listed in Table 1, samples were thawed and homogenized with a FastPrep®-24 Instrument (MP Biomedicals) followed by addition of 2% Saponin (S-1252, Sigma Aldrich) and stored at 4°C O/N after which the supernatant was collected and analyzed for cytokine/chemokine content using a Bio-Plex Pro Mouse Cytokine 23-Plex assay, a Luminex 200 System and Bio-Plex Manager software version 6.0 (BioRad) according to manufacturer’s instructions.

**Table 1.** Cytokine and chemokine protein levels in colon tissue from non-infected and *C. rodentium*-infected WT and Fpr2^−/−^ mice at days 0, 6, and 19 PI.

	Day 0		Day 6		Day 19	
Cytokines	WT	Fpr2^−/−^	WT	Fpr2^−/−^	WT	Fpr2^−/−^
IFN-γ	12.26 (3.71–42.65)†	0.89 (0.07–6.07)	4.66 (2.12–7.61)	18.41 (11.15–25.80)**^##^**	9.8 (6.15–20.39)	26.35 (19.03–33.84)**^&^**
TNF-α	384.5 (217.6–717.2)	230.4 (130.4–483.3)	934.1 (526.4–1129)	295.7 (185.2–367.5)**^#^**	291.9 (270.6–375.7)	264.8 (217.5–303.4)
IL-6	7.69 (6.685–10.11)	1.805 (1.538–9.905)	8.6 (6.57–12.11)	0.77 (0.77–1.853)**^##^**	6.36 (2.54–9.75)	0.77 (0.77–0.77)**^&&^**
IL-17A	14.22 (2.2–43.09)	2.53 (2.11–15.04)	69.96 (43.89–89.91)	56.05 (30.93–87.24)	30.26 (7.023–72.77)	35.82 (25.05–61.49)
IL-12(p40)	792.8 (521.9–1018)	317 (251–362.3)**	424.1 (351.7–1208)	849.3 (522.5–1416)	1144 (775.6–1446)	984.6 (440.4–1669)
IL-1α	5.28 (2.813–7.365)	8.045 (6.075–9.645)	11.43 (7.76–15.44)	19.27 (13.45–33.68)	6.865 (4.955–9.03)	17.91 (12.39–25.98)^&^
IL-1β	293.9 (267.6–341.5)	455.1 (352.6–501.7)*	381.1 (359.5–586.4)	842.6 (624.3–1156)**^#^**	727.2 (471.9–873.3)	695.5 (591.2–874.8)
GM-CSF	38.84 (4.38–81.89)	1.75 (1.75–1.75)*	76.8 (53.35–96.82)	15.75 (1.75–111.3)	77.42 (16.91–115.1)	76.8 (1.75–128.9)
G-CSF	0.11 (0.11–0.11)	4.17 (0.11–31.83)	0.11 (0.11–9.52)	6.26 (0.11–32.72)	ND	ND
IL-2	1.83 (1.83–6.55)	1.83 (1.83–1.83)	9.05 (7.045–17.09)	1.83 (1.83–1.83)**^##^**	ND	ND
IL-4	3.75 (3.75–3.75)	29.82 (20.59–35.17)**	3.75 (3.75–5.64)	24.79 (3.75–47.55)	3.75 (3.75–24.65)	31.61 (10.8–61.82)
IL-5	40.08 (17.25–66.38)	79.48 (59.75–109.6)	54.14 (49.48–63.47)	129.6 (49.4–219.1)	70.38 (31.93–350.7)	130.8 (73.94–227.8)
IL-13	597.5 (489.8–645.9)	282.4 (257.8–330)**	739.2 (679.3–739.2)	406.4 (347.5–506.4)**^##^**	568.9 (525.1–642.4)	407.7 (338.5–495) **^&^**
IL-10	22.61 (5.9–79.03)	5.76 (1.665–9.47)	36.83 (21.3–48.33)	19.84 (10.02–28.99)	24.03 (18.4–31.84)	45.44 (35.4–68.6)
Chemokines	**WT**	**Fpr2^−/−^**	**WT**	**Fpr2^−/−^**	**WT**	**Fpr2^−/−^**
KC	102.6 (65.19–135.7)	53.19 (50.02–65.59)*	113.4 (77.12–382)	511.8 (290.1–638.7)	120.1 (92.17–152.4)	81.48 (75.54–196)
Eotaxin	4733 (3626–7371)	550.1 (550.1–918.4)**	6360 (5534–9027)	4850 (3179–5703)**^#^**	4587 (4320–5814)	4482 (2343–5882)
RANTES	872 (367.4–1306)	466.4 (194.4–721.7)	644.5 (582.8–799.3)	329.5 (198.8–531.9)	1553 (1310–1804)	904 (619.3–1444)
MCP-1	571.5 (428–1461)	593.3 (469.6–684.6)	478.8 (443.8–763.9)	1342 (832.5–2926)**^#^**	913.5 (760.8–1291)	997.1 (713.7–1493)
MIP-1α	87.77 (37.93–252.1)	179.6 (103.4–252.6)	37.93 (37.93–195.3)	348.1 (183.5–1084)**^#^**	342.6 (336.4–437.8)	284.1 (206.1–499.9)
MIP-1β	92.76 (81.16–108.6)	60.7 (49.43–85.13)	101.8 (85.6–110)	202.8 (175.1–290.4)**^##^**	125.8 (119.2–130.9)	153.3 (147.5–163.4)**^&^**

The median values (with 25th and 75th percentile) for each cytokine and/or chemokine (in pg/mL) between WT and Fpr2^−/−^ mice were compared at each time-point (n = 4–6). Statistics: Mann–Whitney U test, *Day 0, ^#^Day 6, ^&^Day 19 p < 0.05 and **, ##, && p < 0.005. † indicates that one mouse had a 4.5 fold higher value than the median of the remaining three mice in the group leading to a high median for the group. ND = Not determined. Of note, IL-3, IL-9, and IL-12 (p70) values were below the detection limit and therefore excluded from the table.

### *Mass spectrometric analysis of* o*-glycans*

Cell lysis and protein extraction: Murine colons from *C. rodentium* infected (low dose) WT and Fpr2^−/−^ mice (n = 3) were processed using VDI 12 hand-held homogenizer (VWR International) and a cell lysis buffer containing 100 mM Tris-HCl pH 7.5, 150 mM NaCl, 1 mM EDTA, 1% Triton X-100, 10 mM DTT and protease inhibitor cocktail (Sigma). Samples were incubated in ice for 15 min, followed by homogenizing twice for 1 min with 5 min pause between treatments. Protein extraction was carried out overnight at 4°C by end-over-end rotation, followed by centrifugation for 30 min, 12,000 RPM at 4°C. The supernatant containing the soluble extract was collected and the protein concentrations were measured using NanoDrop 2000 (Thermo Scientific).

Release of *O*-glycans from extracted proteins: Approximately 100 µg of extracted protein samples were dot blotted on PVDF membrane (Millipore). Proteins were stained using Alcian blue stain solution, excised and transferred to separate Eppendorf tubes. *N*-glycans were removed using 10 U *N*-glycosidase F (PNGase F, *Elizabethkingia miricola*, Promega) in 20 µL water/tube by overnight incubation at 37°C. Subsequently, the Alcian blue-stained spots were subjected to reductive β-elimination with 0.5 M sodium borohydride in 50 mM sodium hydroxide for 16 h at 50°C to release the *O*-glycans. The reduction reaction was quenched by the addition of glacial acetic acid to the mixtures and desalted using strong cation exchange resin packed on top of C18 column. The solid-phase extraction removed cations and any protein or peptide component remaining. Excess borate was extracted as methyl esters by repeated evaporation.

Characterization of *O*-glycans: Released *O*-glycans were analyzed by liquid chromatography-tandem mass spectrometry (LC-MS/MS) using a 10 cm X 250 µm i.d. column (in-house), containing 5 µm porous graphitized carbon (PGC) particles (Thermo Scientific, Waltham, MA, USA) connected to an LTQ mass spectrometer (Thermo Scientific). *O*-glycans were eluted using a linear gradient from 0% to 40% acetonitrile in 10 mM ammonium bicarbonate over 40 min at a flow rate of 250 nl/min. Electrospray ionization-mass spectrometry (ESI-MS) was performed in negative ion polarity with an electrospray voltage of 3.5 kV, capillary voltage of −33.0 V, and capillary temperature of 300°C. The following scan events were used: MS full scan (*m/z* 380–2000) and data-dependent tandem MS (MS/MS) scans after collision-induced dissociation (CID) on precursor ions at a normalized collisional energy of 35% with a minimum signal of 300 counts, isolated width of 2.0 *m/z*, and activation time of 30 ms. The data were viewed and manually analyzed using Xcalibur software (version 2.2, Thermo Scientific). Molecular mass, retention time on PGC column, and tandem mass spectra along with in-house tandem mass spectral glycan library were used for structural identification.

### Statistical analysis

All tests were performed using GraphPad Prism (GraphPad Software, version 7.0). Values are expressed as mean ± S.E.M or geometric mean ± interquartile ranges. Comparison of data between control and infected at a specific time-point was made using the unpaired *t*-test or Mann–Whitney U-test. Kruskal–Wallis test followed by Dunn’s multiple comparison test was used to compare data from more than two experimental groups. Refer to figure legends for the specific statistical test used for each comparison.

## Results

### Epithelial Fpr2 staining intensity increase during *C. rodentium* infection

**Figure 1.**
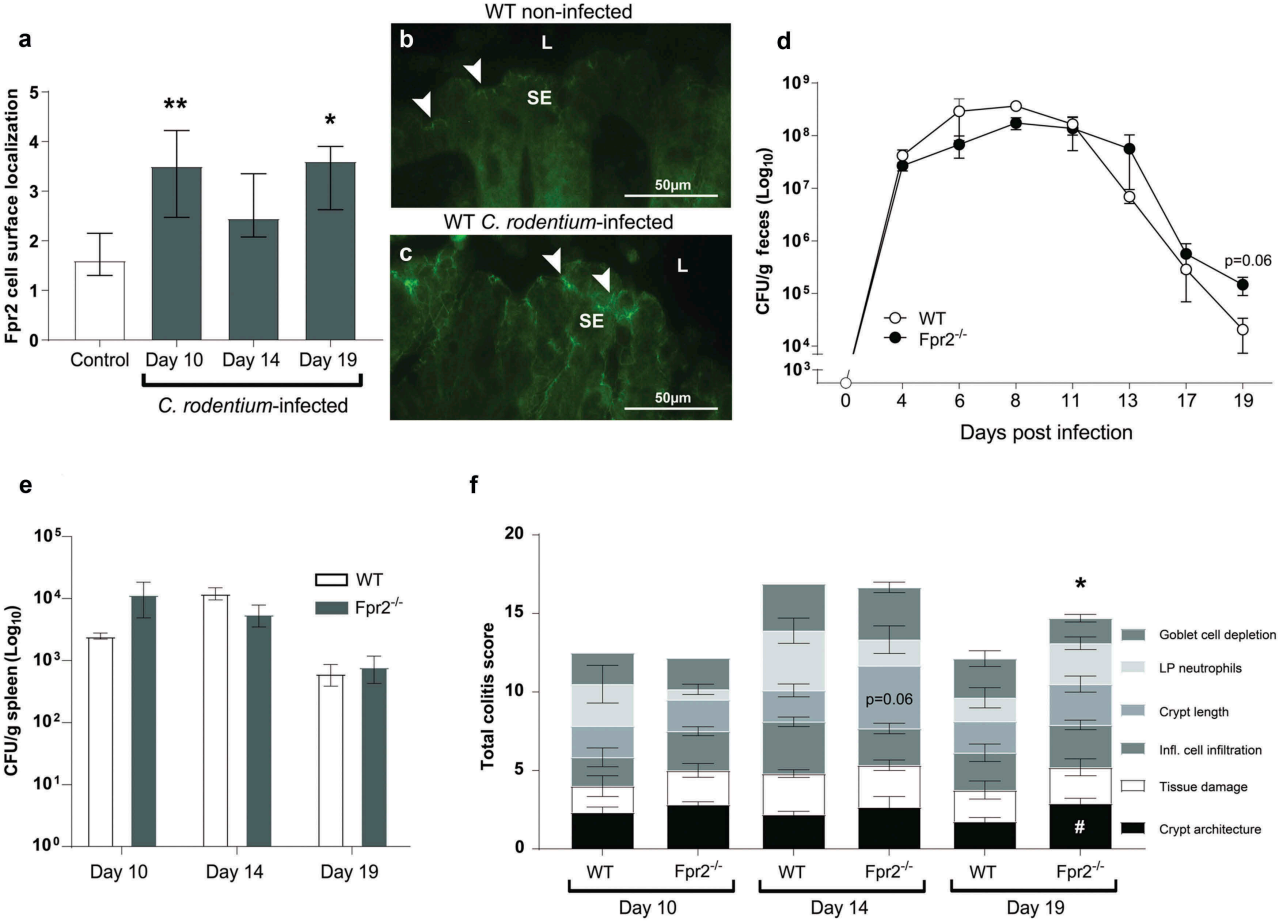
Fpr2 cell surface localization and infection parameters in WT and Fpr2^−/−^ mice following infection with *C. rodentium*. (a) Fpr2 localization at the colonic epithelial cell surface in infected (day 10, 14 and 19 PI) and non-infected mice. n = 4–9 mice/time-point. (b and c) Representative images of Fpr2 in distal colon of (b) non-infected and (c) infected WT mice at day 10 PI (arrowheads = Fpr2, L = lumen and SE = surface epithelium. Images were captured using a 40x objective. (d) Fecal CFU count of WT and Fpr2^−/−^ mice inoculated with *C. rodentium* 5 × 10^9^ CFU. (e) Spleen CFU counts of WT and Fpr2^−/−^ mice at day 10, 14 and 19 PI. (f) Total colitis score of WT and Fpr2^−/−^ mice at day 10, 14 and 19 PI. The score represents the sum of the following individual parameters with a maximum score of 4 for each; crypt architecture, crypt length, goblet cell depletion, number of lamina propria neutrophils, inflammatory cell infiltration, and epithelial tissue damage and ulceration. n = 4–9 mice per group. Statistics: (a) Kruskal–Wallis test followed by Dunn’s multiple comparison test, *p < 0.05, **p < 0.001 vs control, (f) Mann–Whitney U-test, #p < 0.05, crypt architecture, *p < 0.05, total colitis score WT vs Fpr2^−/−^ at each time point. Error bars; (a) median interquartile range, D, E, and F) mean S.E.M.

To analyze effects of *C. rodentium* infection on epithelial Fpr2 localization we selected three time-points based on earlier work: day 10 (peak of *C. rodentium* density in feces: ~10^8^ CFU/g), day 14 (start of decrease in bacterial density: ~10^7^ CFU/g) and day 19 (close to clearance of infection: ~10^2^–10^4^ CFU/g) PI [28,29]. Epithelial Fpr2 was detected in non-infected wild type (WT) mice and the intensity of Fpr2 in epithelial cell junctions and apical localization increased during *C. rodentium* infection, especially at day 10 and 19 PI (p < 0.05, Figure 1(a–c)).

### Fpr2^−/−^ mice recover more slowly from C. rodentium infection than WT mice

WT and Fpr2^−/−^ mice infected with a conventional dose (5 x 10^9^ CFU) showed a similar pathogen burden until day 17 PI (Figure 1(d)); however, at day 19 PI Fpr2^−/−^ mice tended to have 10-fold higher fecal *C. rodentium* CFU counts (p = 0.06, Figure 1(d)). No difference in the number of *C .rodentium* in the spleen was observed between groups (Figure 1(e)). In line with these results, the colitis scores were similar at day 10 and 14 PI in WT and Fpr2^−/−^ mice (Figure 1(f)), whereas at day 19 PI the total colitis score was higher in Fpr2^−/−^ than in WT mice (p < 0.05, Figure 1(f)).

### Fpr2^−/−^ mice have increased susceptibility to C. rodentium colonization and develop more severe colitis in response to low dose infection

**Figure 2.**
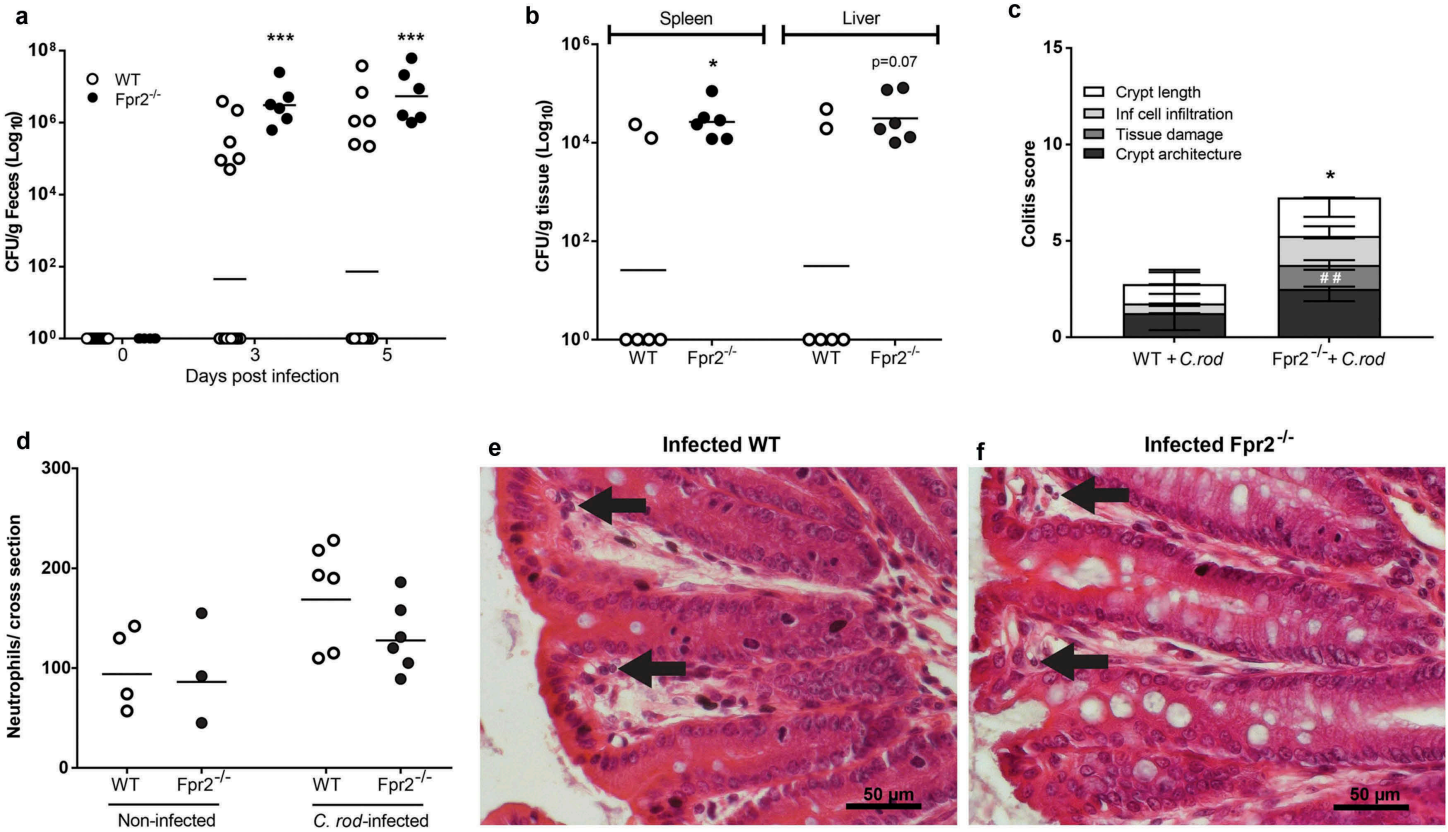
*C. rodentium* density, translocation and colitis score during early infection of WT and Fpr2^−/−^ mice infected with a low dose of *C. rodentium*. (a) Fecal CFU count of Fpr2^−/−^ mice and their WT controls inoculated with 5 × 10^7^ CFU of *C. rodentium*. All Fpr2^−/−^ mice became infected in contrast to only 6 out of 20 WT mice (n Fpr2^−/−^ = 6, WT = 20). (b) Of the mice that became infected, the level of *C. rodentium* in the spleen was higher in Fpr2^−/−^ compared to WT mice at day 6 PI and a similar trend was observed for *C. rodentium* in the liver (n = 6). (c) Colitis score of H&E stained distal colon sections from WT and Fpr2^−/−^ mice at day 6 PI (n = 6, maximum score 4). Non-infected WT mice from the inoculated group (n = 14) were excluded, as they did not show signs of colitis (scores were similar to that of non-infected controls <1). (d) Manual neutrophil count for entire cross-sections of H&E stained distal colon specimens from non-infected and infected WT and Fpr2^−/−^ mice (n non-infected = 3–4, infected = 6). (e and f) Representative H/E stained distal colon samples from infected WT (e) and Fpr2^−/−^ (f) mice (arrows highlighting neutrophils in lamina propria). Statistics: (a–d) Mann–Whitney U-test, *p < 0.05, ##p < 0.001, and ***p < 0.0005. Error bars; (a, b, and d) Geometric mean and (c) median with interquartile range.

To assess whether Fpr2^−/−^ mice are more susceptible to pathogen colonization than WT mice, we used a *C. rodentium* inoculum dose aimed to infect only a low proportion of WT mice (5 × 10^7^ CFU/mouse), which is 100 fold lower than the dose used for the mice in Figure 1 and 10–100 fold lower than the standard dose [30,31], and harvested mice at day 6 PI. Our results showed that 100% of the Fpr2^−/−^ mice became colonized, in contrast to only 30% of the WT mice (p < 0.0005, Figure 2(a)). Among colonized animals, colitis scores and *C. rodentium* CFUs in the spleen were higher in Fpr2^−/−^ compared to WT mice (p < 0.05, Figure 2(b and c)) and a similar trend was found in the liver CFU count (p < 0.07, Figure 2(b)). Among colonized animals, the number of neutrophils in the tissue tended to be slightly higher in infected WT mice as compared to Fpr2^−/−^ mice (p = 0.14, Figure 2(d)). Inoculated WT mice that did not become infected had similar colitis scores as non-infected WT and Fpr2^−/−^ controls (colitis score <1).

### More bacteria are in direct contact with colonic epithelial cells in Fpr2^−/−^ than in WT mice

**Figure 3.**
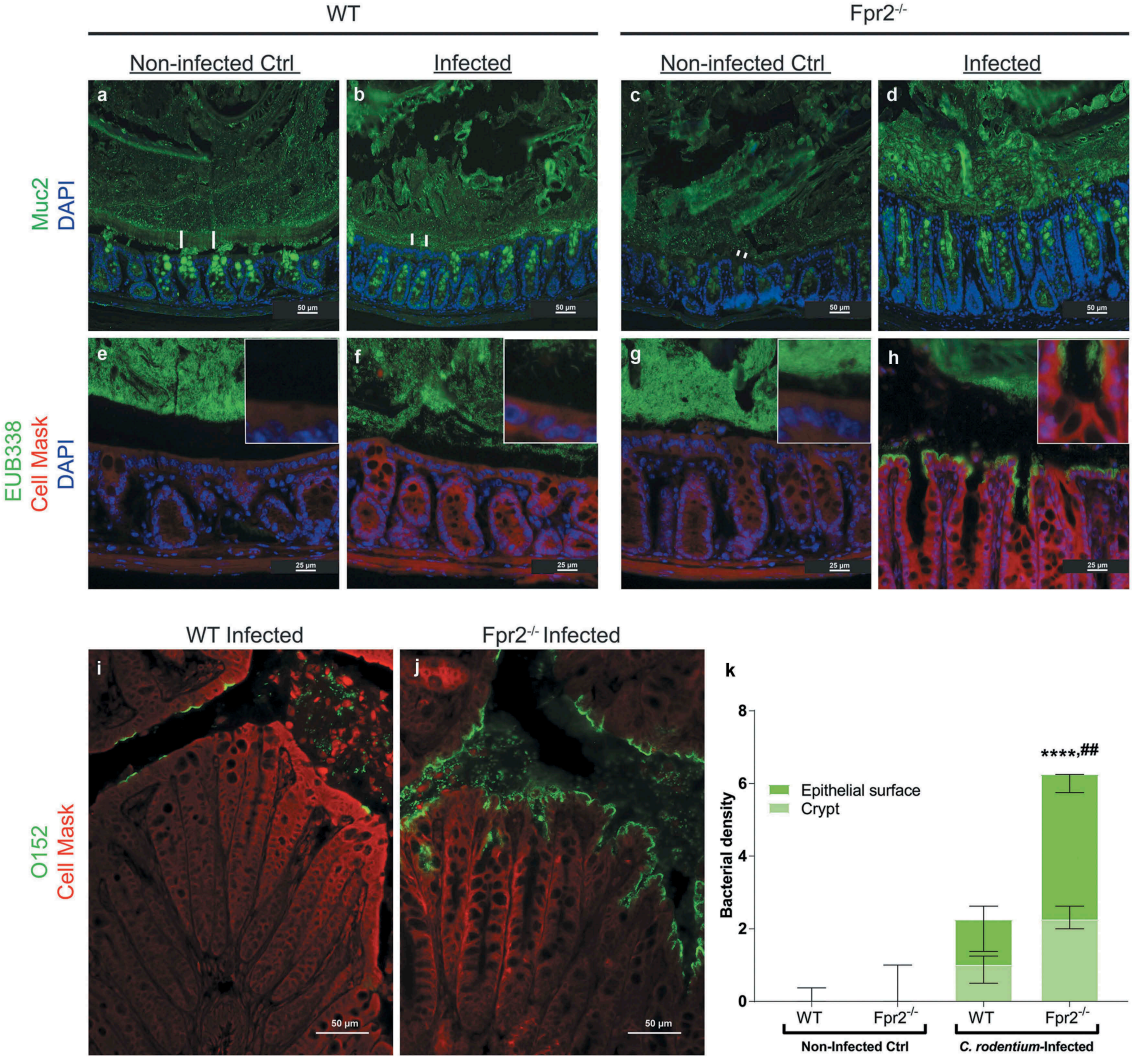
Mucus layer organization and bacterial localization in the distal colon of WT and Fpr2^−/−^ mice day 6 after low dose *C. rodentium* infection. (a–d) Representative immunofluorescence images of MUC2 (green) and nuclei (DAPI, blue) showing the presence of an inner mucus layer with a striated appearance (indicated by ||) separating luminal contents from the epithelium in non-infected control (a) and infected WT (b) mice, respectively. This was in contrast to Fpr2^−/−^ mice in which a clearly striated inner mucus layer was absent regardless of their infection status (c and d). (e–h) Bacterial localization (EUB338 probe hybridizing with eubacteria including *C. rodentium*, green) at the epithelium and crypts (outlined by Cell Mask, red) in the distal colon, white boxes show close-ups of the surface epithelium for each genotype/image. In WT non-infected control mice, the vast majority of bacteria were at a distance from the epithelial surface (e) whereas a low amount of bacteria were in contact with the epithelium in the infected WT mice (f) and Fpr2^−/−^ non-infected control mice (g) and a large amount of bacteria were present in close contact with the epithelium in infected Fpr2^−/−^ mice (h). (i and j) *C. rodentium* localization in infected WT (i) and Fpr2^−/−^ (j) mice detected using an antisera targeting the O152 antigen (also found in *C. rodentium*). (k) Quantification of bacterial density in close proximity of the epithelial surface or colonic crypts in *C. rodentium* infected WT and Fpr2^−/−^ mice. n = 5–6. Statistics: (k) Kruskal–Wallis test followed by Dunn’s multiple comparison test, ****p < 0.0001 vs WT non-infected control, ^##^p < 0.005 vs WT + *C. rod*. Error bars; Median interquartile range.

A bacteria free, striated, organized mucus layer that appeared relatively thick was present in non-infected WT mice (Figure 3(a and e)). In the infected WT mice, this layer appeared thinner, less organized and contained bacteria (Figure 3(b and f)), this in line with previously published results from explant specimens demonstrating a decrease in mucus thickness during early infection [28]. In non-infected Fpr2^−/−^ mice, bacteria were present close to the epithelium with epithelial cells and the mucus layer appeared thin/scant (Figure 3(c and g)). At day 6 PI, the mucus layer in Fpr2^−/−^ mice contained bacteria and appeared thick but disorganized, and a large amount of *C. rodentium* were in direct contact with the epithelial cells (Figure 3(d, h, j, and k)). More bacteria, including *C. rodentium*, were found in close contact with the epithelium of infected Fpr2^−/−^ mice than non-infected (p < 0.0001) and infected WT mice (p < 0.005, Figure 3(i–k)). These findings suggest that the thin disorganized inner mucus layer in Fpr2^−/−^ mice allows more bacteria including pathogens to gain access to the epithelial surface.

### Higher density of intercrypt mucus but decreased mucin production and speed of transport en route exocytosis in infected Fpr2^−/−^ mice

**Figure 4.**
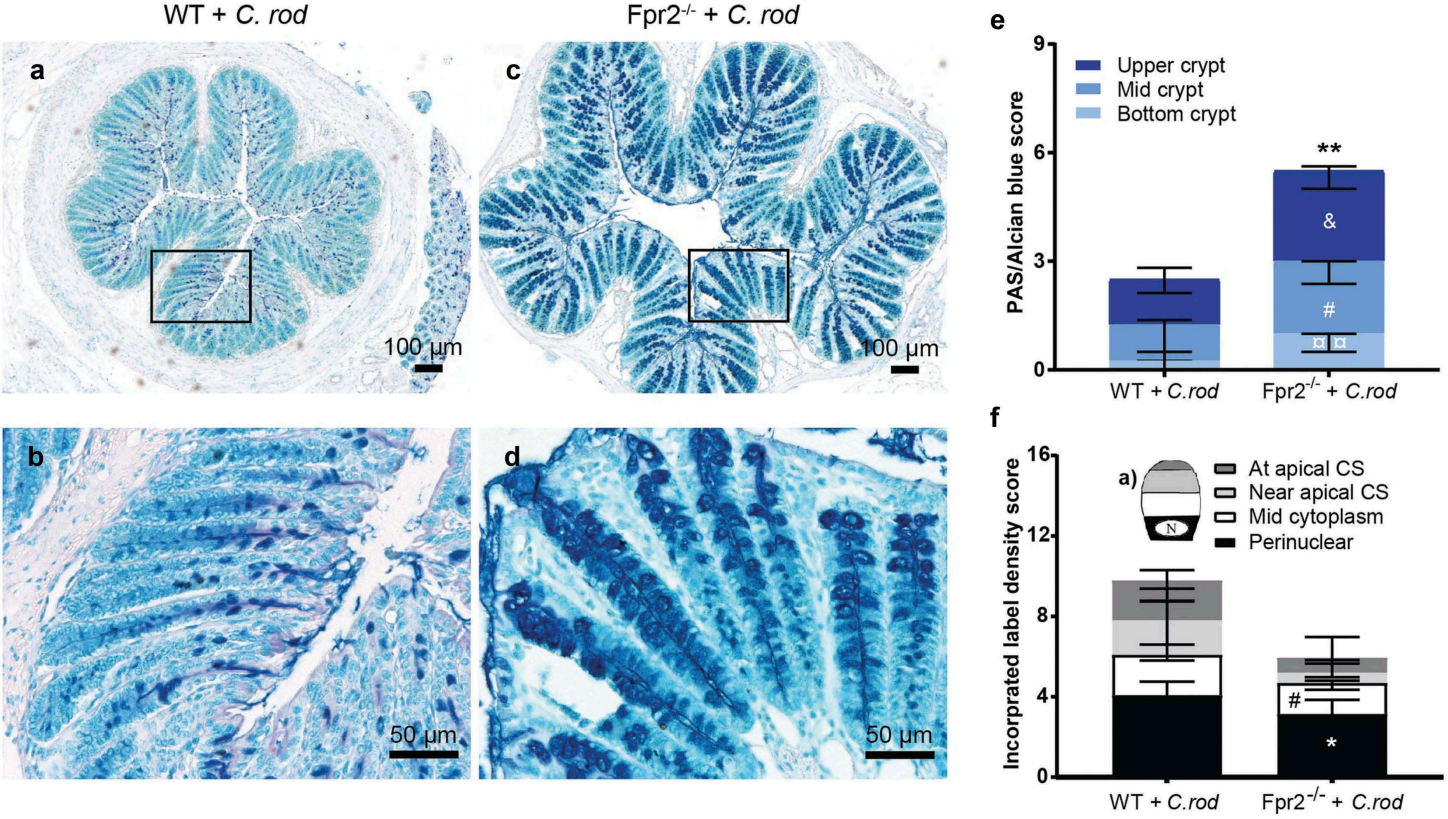
Mucin density in crypts and goblet cell mucin production and transport en route secretion during early *C. rodentium* infection. (a–d) Representative PAS/Alcian blue-stained distal colon samples from WT and Fpr2^−/−^ mice infected with 5 × 10^7^ CFU of *C. rodentium* at day 6 PI using 10x and 20x objective from infected WT (a and b) and Fpr2^−/−^ (c and d) mice. (e) Density score of mucus along the crypts separated into; bottom-, mid- and upper-crypt. Each compartment of the crypt received a score from 0 to 3 based on density, ^&^p < 0.05 upper crypt, ^#^p < 0.05 mid crypt, ¤¤p < 0.005 bottom crypt, **p < 0.005 total score. n = 5–6 mice. (f) Blinded visual semi-quantification of the intensity of incorporated GalNAz in goblet cells (n = 5–6 mice). (a) Schematic representation of a goblet cell and the four compartments analyzed for GalNAz incorporation, N refers to the nucleus. (f) Each location received a score of 0–3 based on intensity, *p < 0.05 perinuclear, ^#^p < 0.05 mid cytoplasm. Mann–Whitney U-test. Error bars; median with interquartile range.

To determine the quantity of secreted mucin glycoprotein along the crypts in the distal colon, we quantified PAS/Alcian blue-stained tissue sections and found more mucin at each compartment of the crypt in infected Fpr2^−/−^ compared with WT mice (bottom p < 0.05, mid p < 0.05 and upper crypt p < 0.005, Figure 4(a–e)). To investigate if the apparent larger amount of mucin found in Fpr2^−/−^ mice were due to increased mucin production, we metabolically labeled the mucins. The azide-modified galactosamine GalNAz incorporate into the core region of mucin *O*-glycans and can be used to analyze mucin production and transport [32–34]. Mass spectrometric analysis verified that 97–99% of the GalNAc residues of the murine colonic mucins from *C. rodentium* infected mice were in the core region, suggesting that the few percent in the terminal region would not affect the incorporation rate of label to a notable extent (median WT 2.6%, median Fpr2^−/−^ 1.6%). Newly synthesized mucins can be visualized in the supranuclear compartment of the cell 1 h after injection [33] and reach the colonic lumen in 6–8 h [35]. We compared the quantity and cellular location of labeled mucins in goblet cells of infected WT and Fpr2^−/−^ mice at day 6 PI, 3 h post-GalNAz injection because by that time the mucins are inside the cells and processing artifacts can be avoided. We scored for location (perinuclear, mid cytoplasm, near apical surface, and at apical surface) and intensity of GalNaz labeled mucins at each compartment of the surface goblet cells (Figure 4(f)). The metabolic label was predominantly found in the perinuclear area of goblet cells of both groups (Figure 4(f)). The main difference between Fpr2^−/−^ and WT mice at day 6 PI was a decreased amount of labeled mucins in the perinuclear (p < 0.05) and mid cytoplasmic (p < 0.05) region (Figure 4(f)). This suggests that in spite of more mucins being present along the crypt, the production of mucins in the distal colon is slower in Fpr2^−/−^ than WT mice.

### Mucin glycans from *C. rodentium* infected Fpr2^−/−^ mice are longer and more sialylated than those from WT mice

**Figure 5.**
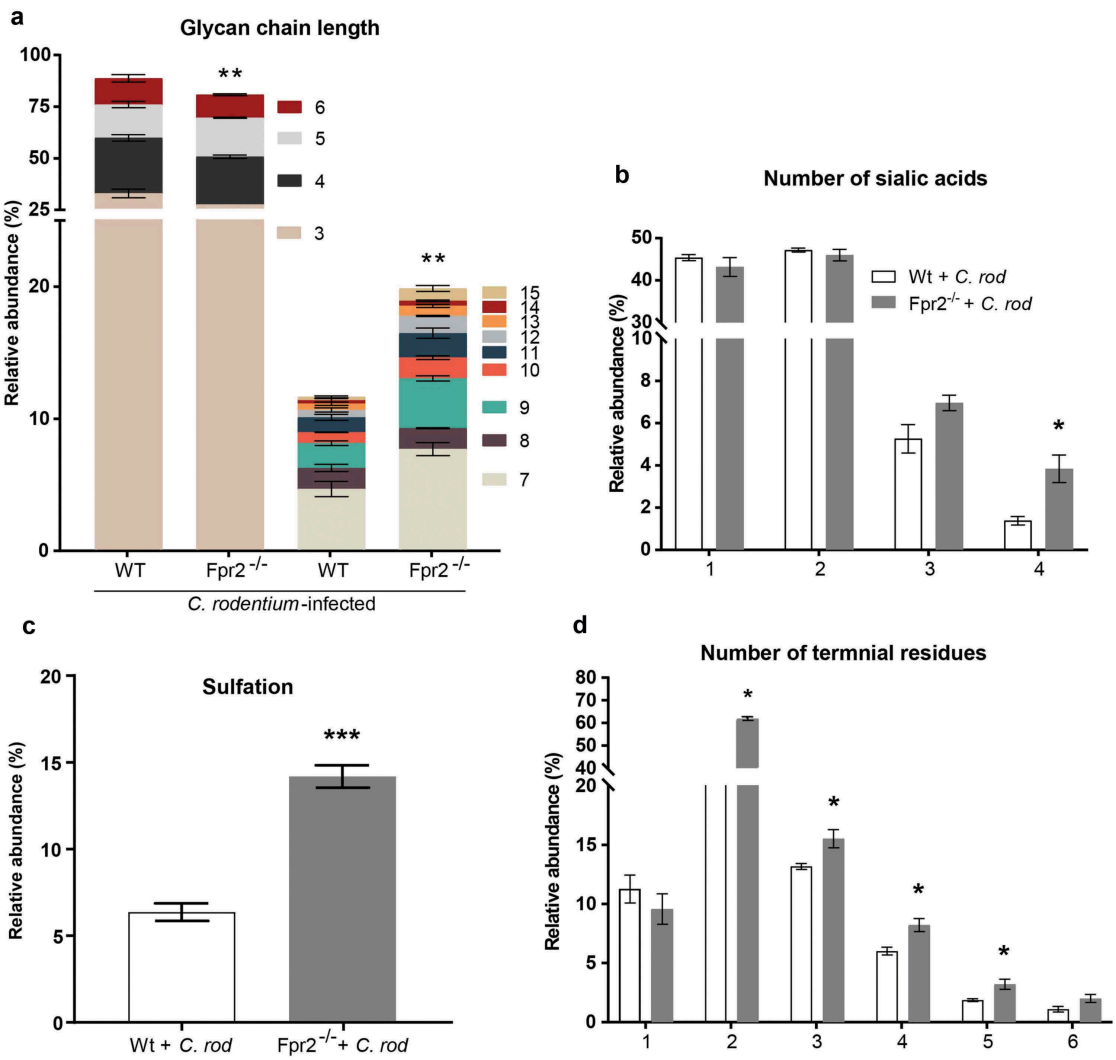
Mucin *O*-glycans from *C. rodentium* infected WT and Fpr2^−/−^ mice during early colonization characterized by mass spectrometry. (a) Size distribution (number of monosaccharide residues/glycan) derived from the overall compositions of *O*-glycans. (b) Relative abundance (%) of *O*-glycans containing 1–4 sialic acid residues. (c) relative abundance (%) of sulfated structures detected on GlcNAc. (d) Relative abundance (%) of terminal residues on *O*-glycans. n = 3 mice. Statistics: Unpaired t-test, (a) glycan chain lengths of 3–6 pooled and 7–15 pooled. Error bars; Mean S.E.M.

Mucin *O*-glycans from *C. rodentium* infected Fpr2^−/−^ and WT mice were characterized by liquid chromatography-tandem mass spectrometry (LC/MS). In total, 78 non-redundant *O*-glycans were characterized by colonic mucins from three WT and three Fpr2^−/−^ mice. Out of these 78 defined *O*-glycans, 66 were identified in both WT and Fpr2^−/−^ mice. The characterized *O*-glycans ranged from neutral glycans containing fucosylation and acidic glycans with *N*-acetylneuraminic acid (NeuAc), *N*-glycolylneuraminic acid (NeuGc) and sulfation. Short (≤6 residues) mucin *O*-glycans were more abundant among WT than Fpr2^−/−^ mice (p < 0.005), whereas the opposite was true for large glycan structures (7–15 residues long, Figure 5(a)). *O*-glycans from both WT and Fpr2^−/−^ mice were highly sialylated and we identified up to four sialic acid residues in branched *O*-glycans. In addition, the relative abundance of *O*-glycans carrying four sialic acid residues was threefold higher among infected Fpr2^−/−^ compared to WT mice (p < 0.05, Figure 5(b)). Sulfated structures that were identified were either on galactose or *N*-acetylglucosamine (GlcNAc), and the level of sulfation was higher (p < 0.001, Figure 5(c)) in Fpr2^−/−^ mice as compared to WT mice. The *O*-glycan structures identified in WT and Fpr2^−/−^ mice were predominantly branched, carrying up to six terminal residues. The relative abundance of *O*-glycan structures with more than three terminal residues, i.e. the highly branched *O*-glycans, was higher in Fpr2^−/−^ mice as compared to the WT mice (Figure 5(d)). These results support that slow mucin biosynthesis occurs in Fpr2^−/−^ mice as shown by the metabolic labeling experiment (Figure 4(f)), as larger and more complex glycans are in line with longer time spent in the biosynthesis machinery in the Golgi.

### The cytokine response elicited during *C. rodentium* infection differs between WT and Fpr2^−/−^ mice

To identify the cytokine environment at time-points related to susceptibility to – and clearance of – infection and reduced mucin production, we investigated a panel of 23 cytokines and chemokines at the protein level in distal colon samples from WT and Fpr2^−/−^ mice from day 0, 6 (low dose of *C. rodentium*), and 19 PI (conventional dose of *C. rodentium*). At day 6 PI, WT mice showed an increase in the inflammatory cytokines: IL-1β (p < 0.05), IL-17A (p < 0.05), and IL-2 (p < 0.05) compared to their non-infected WT controls (Table 1). The protein levels of the vast majority of the cytokines found in the panel were similar among WT and Fpr2^−/−^ mice. However, higher levels of Tumor necrosis factor α (TNF-α) (p < 0.05), IL-6 (p < 0.005), IL-2 (p < 0.005) and IL-13 (p < 0.005) were detected in WT compared to Fpr2^−/−^ mice at day 6 PI (Table 1). Moreover, at that time-point, the majority of chemokines and chemotactic cytokine levels increased in Fpr2^−/−^ mice (Macrophage Chemotactic Protein (MCP)-1 (p < 0.05), Macrophage Inflammatory Protein (MIP)-1α (p < 0.05) and MIP-1β (p < 0.005) and a similar trend observed for KC (p < 0.052)) in relation to WT mice. By day 19 PI the level of both Interferon-γ (IFN-γ) (p < 0.05) and MIP-1β (p < 0.05) remained elevated with an increase in IL-1α (p < 0.05) (Table 1). The difference in the cytokine response between WT and Fpr2^−/−^ mice may contribute to the altered mucin biosynthesis.

### More *C. rodentium* and EPEC translocate across the *in vitro* mucosal surface to the basolateral compartment following FPR2 inhibitor treatment

**Figure 6.**
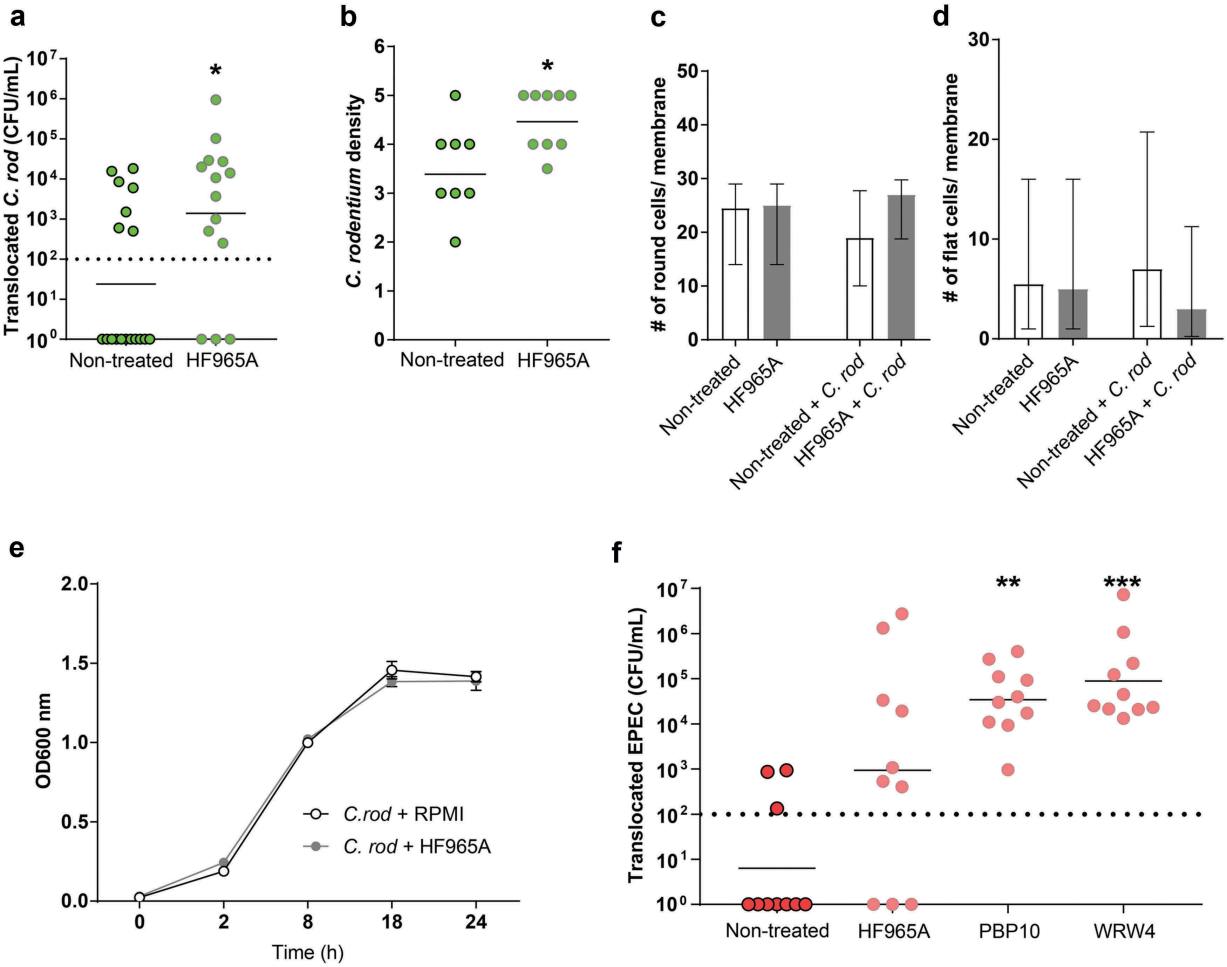
Effect of FPR inhibitor on human *in vitro* colonic mucosal surfaces during *C. rodentium* infection. (a) CFU in the basolateral compartment (i.e., amount of bacteria that crossed the *in vitro* mucosal surface) of non-treated and FPR2 inhibitor (HF965A) treated *C. rodentium* infected *in vitro* mucosal surfaces at 8 h PI (dotted line highlights minimum detection limit, n = 14–18). (b) Semi-quantification of bacterial density in *C. rodentium* infected non-treated and treated *in vitro* mucosal surfaces (n = 8–9). (c and d) The number of round (c), and flat (d) cells found along the entire *in vitro* mucosal surface section with/without treatment and with/without infection (n = 3–8). (e) *C. rodentium* growth in the presence or absence of HF965A measured for a duration of 24 h at OD 600 nm (n = 6). (f) CFU in the basolateral compartment of non-treated and FPR2 inhibitor-treated EPEC infected *in vitro* mucosal surfaces at 5 h PI (dotted line highlights minimum detection limit, n = 10). Statistics: (a and b) Mann–Whitney U-test, *p < 0.05 vs non-treated. (f) Kruskal–Wallis test followed by Dunn’s multiple comparison test, **p < 0.005, ***p < 0.0001 vs non-treated. Error bars; (a, b and f) geometric mean, (c and d) median interquartile range and (e) Mean S.E.M.

Next, the role of FPR2 in bacterial mucosal trans-migration was studied using *in vitro* mucosal surfaces based on human colonic HT29 MTX-E12 cells forming polarized goblet cells, tight junctions and a three-dimensional architecture with apical mucus secretion [26]. We found that more *C. rodentium* translocated across *in vitro* mucosal surfaces into the basolateral compartment when pre-treated with the FPR2 inhibitor HF965A than across untreated ones (p < 0.05, Figure 6(a)). Furthermore, more *C. rodentium* were present intracellularly and in between cell junctions in *in vitro* mucosal surfaces when treated with the FPR2 inhibitor (p < 0.05, Figure 6(b)). Assessments of entire membrane sections did not indicate any morphological alterations regardless of inhibitor treatment and in combination with *C. rodentium* for 24 h (Figure 6(c and d)). To rule out any effect of the inhibitor on the growth of *C. rodentium* we carried out a growth assay in the presence and absence of the FPR2 inhibitor and found that both growth curves coincided throughout the 24-h incubation, demonstrating that the differences in invasion/translocation were not due to effects on *C. rodentium* growth (Figure 6(e)). To verify the role of epithelial FPR2 in bacterial translocation, we infected the *in vitro* mucosal surfaces with EPEC, using two additional FPR2 inhibitors and found that PBP10 (p < 0.005) and WRW4 (p < 0.0001) treated *in vitro* mucosal surfaces allowed more EPEC to translocate to the basolateral compartment compared to untreated ones, and a similar trend was observed with the FPR2 inhibitor HF965A (Figure 6(f)). The degree of increased permeability after treatment with HF965A was similar in the *C. rodentium* and EPEC experiments. However, the multi-comparison correction in the EPEC experiment rendered the difference above the limit for statistical difference (Kruskal–Wallis ANOVA: p = 0.12, Mann–Whitney U-test: p = 0.03). As EPEC is more aggressive and grows faster than *C. rodentium*, we collected the CFU data at 5 h PI, before the monolayer was completely destroyed by the pathogen. These *in vitro* results further indicate that the lack of functional FPR2 in colonic epithelial cells increases bacterial translocation and plays an important role in limiting bacterial dissemination.

## Discussion

In this study, we demonstrated that infection of WT mice with *C. rodentium* caused an increase in apical Fpr2 localization in colonic epithelial cells. Inoculating Fpr2^−/−^ mice with a *C. rodentium* dose designed to infect all animals resulted in bacterial titers and colitis scores similar to those of WT mice at day 10 and 14 PI, although the colitis healed slower in Fpr2^−/−^ than WT mice. However, Fpr2^−/−^ mice had increased susceptibility to *C. rodentium* colonization: among the 100% of the Fpr2^−/−^ and 30% of the WT mice that became colonized, Fpr2^−/−^ mice developed more severe colitis and more *C. rodentium* were in contact with the epithelial cells in response to low dose infection. In line with the larger amount of *C. rodentium* detected in the spleen in Fpr2^−/−^ mice, more *C. rodentium* translocated across the *in vitro* mucosal surface to the basolateral compartment following FPR2 inhibitor treatment. Although the Fpr2^−/−^ mice had an increased quantity of mucin in goblet cells and plenty of mucus covering the epithelium, they lacked the striated inner mucus layer present in WT mice. Decreased mucus production and different glycosylation may contribute to the defect inner mucus layer in Fpr2^−/−^ mice.

Bacteria start their protein synthesis with an N-formyl-Met and the formyl group has to be removed in order to process mature proteins [22]. Thus, the release of formyl peptides with different lengths and amino acid sequence is a hallmark of bacterial metabolism. In line with this, both FPR1 and FPR2 recognizing formyl peptides have been identified from bacteria including *Escherichia coli* and *Staphylococcus aureus* [23–25]. Based on this, it is likely that *C. rodentium* release formyl peptides recognized by the innate immune system through FPR2. We confirmed previous reports on the presence of Fpr2 in intestinal epithelial cells [17,36]. Additionally, we observed increased apical Fpr2 localization after *C. rodentium* infection. It has been shown that mice lacking Fpr2 or genes that encode anti-inflammatory Fpr2-binding proteins are more susceptible to DSS-induced colitis [17,37]. Complementary to this, we demonstrated that Fpr2^−/−^ mice were also more susceptible to colonization by a low dose of *C. rodentium* than WT mice. We selected a dose, 10–100-fold lower than recommended for infection of 100% of the mice [30,31], with the aim to infect only a subset of the WT animals. Under natural conditions, the body is frequently exposed to pathogenic bacteria, although infection after exposure is relatively rare. The body has a range of defense mechanisms that can fend of low doses of pathogens. The doses conventionally used for infection experiments are designed to be high enough to override these barriers to ensure that 100% of the mice become infected, enabling studies of events occurring during infection using a limited number of mice. In contrast, when the aim of an experiment is to investigate if barriers to colonization are decreased, a dose designed to colonize only a subset of the control group better points to decreased barrier functions, as demonstrated by the low dose infection experiment.

Among colonized mice, Fpr2^−/−^ mice had more bacteria in direct contact with the epithelium and in the spleen, suggesting compromised barrier functions. The barrier provided by the mucus layer appeared defect: the inner striated mucus layer that keeps bacteria away from the epithelial surface in the WT mice was absent or disorganized in the Fpr2^−/−^ mice. The outer mucus layer, where the microbial flora reside, is degraded by commensal microbes [2]. Therefore, this area requires constant renewal by goblet cells in order to maintain the inner mucus layer that keeps bacteria from getting in direct contact with the underlying mucosa. Several factors may contribute to the diminished inner mucus layer in the Fpr2^−/−^ mice. Firstly, the reduced mucin production may lead to a mucin-degrading activity of the microflora that acts faster than the mucus replenishment. Secondly, the changed glycosylation likely alters the microflora, a factor that both can affect the mucus layer production and degradation rate [38]. Finally, the increased negative charge of the mucin *O*-glycans increases inter-mucin repulsion, potentially resulting in less efficient packaging in mucus granules as well as between mucins in the mucus layer, in line with the more loose and thick appearance in the stained tissue sections. The increased amount of mucin detected in the tissue of Fpr2^−/−^ mice cannot be explained by the rate of mucin synthesis, since mucin production was impaired in these mice. Possibly, the increased sialylation protects the mucin from bacterial degradation, leading to mucus accumulation.

We have previously observed that the cytokine profile changes during the course of *C. rodentium* infection and is able to regulate mucus thickness and quality affecting bacterial localization relative to the epithelium in the distal colon of WT mice [39]. FPR2/Fpr2 is able to respond to both bacterial and endogenously released formylated peptides, to exert both pro- and anti-inflammatory actions [40]. In addition, recent data show that some ligands trigger biased signaling and induce selective functional responses [41,42]. Several FPR agonists promote inflammation resolution in *in vivo* disease models [43]. Overall, relatively similar inflammatory cytokine responses were observed in the two genotypes at day 6 and 19 PI indicating that Fpr2^−/−^ mice do not lack a protective response. However, the lower levels of TNF-α, IL-2, IL-6, and IL-13 in Fpr2^−/−^ mice during early infection might account for alterations related to mucus production and goblet cell function, decreased early host defense, and increased mucosal inflammation and pathogen dissemination, since cytokines affect these aspects [39,44–47].

We found higher numbers of *C. rodentium* translocated from the colon to the spleen in Fpr2^−/−^ mice, which indicate increased epithelial permeability in the absence of this receptor. Epithelial permeability to dextran has been reversed in DSS treated mice following the administration of an Fpr2 agonist highlighting the importance of Fpr2 in maintaining a stable epithelial barrier [48]. Since FPR2/Fpr2 is also highly expressed by phagocytic cells and is involved in their recruitment to inflamed tissues [49,50] the higher titers of *C. rodentium* found in the spleen of Fpr2^−/−^ mice may in part be due to poor immunosurveillance of invading bacteria in extraintestinal tissues early during infection, before any *C. rodentium*-specific IgG antibodies are produced to support clearance of the pathogens [51]. However, pre-treatment with an FPR2 inhibitor allowed more *C. rodentium* to translocate across polarized mucus producing *in vitro* mucosal surfaces than across untreated ones, suggesting that also FPR2 signaling in the epithelial cells participates in limiting translocation of pathogens across the epithelial lining. Epithelial cell restitution is of paramount importance to recover the mucosa following injury or infection. Mitochondrial proteins from damaged cells and bacterial formylated peptides have been shown to enhance wound closure in cultured intestinal epithelial cells by activating FPRs [14,52]. Also, mice deficient in AnnexinA1, an anti-inflammatory protein that binds to Fpr2, fail to recover the damaged epithelium following DSS-induced colitis in mice [37]. The contribution of FPR2 to wound healing and anti-inflammatory effect may explain why WT mice recover faster from the infection-induced tissue injury than Fpr2^−/−^ mice as evident at day 19 PI in the current study. Knowledge on the mechanisms that regulate mucosal barrier functions may enable the development of therapeutic regimens against infection and inflammatory bowel disease. Enhancing the activity of FPR2 by selective agonists may have therapeutic benefits to protect against infection or aid mucosal healing; however, further studies are needed to support this.

In conclusion, we have demonstrated a role for FPR2/Fpr2 in the defense against colonization with human and murine pathogenic bacteria. The lack of Fpr2 renders mice more susceptible to *C. rodentium* infection due to decreased colonic barrier function comprising both the mucus layer and the epithelial cell layer barriers. The present work supports a model in which FPR2/Fpr2 governs epithelial defenses against infection, including the mucus layer, and restitution of damaged mucosal surfaces.

## Abbreviations

BSA: Bovine serum albumin
EDTA: Ethylenediaminetetaacetic acid
DTT: Ditiotreitol
TAMRA: Tetramethylrhodamine
GalNAz: N-azidoacetylgalactosamine
GlcNAc: N-acetylglucosamine
NeuAc: N-acetylneuraminic acid
NeuGc: N-glycolylneuraminic acid
LC-MS: Liquid chromatography-mass spectrometry
C. rodentium: Citrobacter rodentium
E. coli: Escherichia coli
EPEC: Enteropathogenic Escherichia coli
EHEC: Enterohaemorrhagic Escherichia coli
A/E: Attaching and effacing
CFU: Colony forming unit
PI: Post infection
WT: Wild-type
GI: Gastrointestinal
DSS: Dextran-sulfate sodium
FPR: Formyl peptide receptor
FPR2-/-: Formyl peptide receptor 2 knock-out
GPCR: G-protein coupled receptor
IL: Interleukin
TNF-α: Tumor necrosis factor alfa
IFN-γ: Interferon gamma
MCP-1: Macrophage chemotactic protein-1
MIP: Macrophage inflammatory protein
H/E: Hematoxylin/eosin
PAS: Periodic acid Shiff
LB: Luria Bertani broth
FBS: Fetal bovine serum

## References

[1] McGuckin MA, Linden SK, Sutton P, et al Mucin dynamics and enteric pathogens. Nat Rev Microbiol. 2011;9(4):265–278. .Epub 2011/03/17; nrmicro2538 [pii]; PubMed PMID: 21407243 21407243 10.1038/nrmicro2538

[2] Johansson ME, Phillipson M, Petersson J, et al The inner of the two Muc2 mucin-dependent mucus layers in colon is devoid of bacteria. Proc Natl Acad Sci U S A. 2008;105(39):15064–15069. .PubMed PMID: 18806221; PubMed Central PMCID: PMC2567493 18806221 10.1073/pnas.0803124105 PMC2567493

[3] McGuckin MA, Every AL, Skene CD, et al Muc1 mucin limits both Helicobacter pylori colonization of the murine gastric mucosa and associated gastritis. Gastroenterology. 2007;133(4):1210–1218. .PubMed PMID: 17919495 17919495 10.1053/j.gastro.2007.07.003

[4] Bergstrom KS, Kissoon-Singh V, Gibson DL, et al Muc2 protects against lethal infectious colitis by disassociating pathogenic and commensal bacteria from the colonic mucosa. PLoS Pathog. 2010;6(5):e1000902 .PubMed PMID: 20485566; PubMed Central PMCID: PMCPMC2869315 20485566 10.1371/journal.ppat.1000902 PMC2869315

[5] McAuley JL, Linden SK, Png CW, et al MUC1 cell surface mucin is a critical element of the mucosal barrier to infection. J Clin Invest. 2007;117(8):2313–2324. .PubMed PMID: 17641781; PubMed Central PMCID: PMCPMC1913485 17641781 10.1172/JCI26705 PMC1913485

[6] Santos AS, Finlay BB. Bringing down the host: enteropathogenic and enterohaemorrhagic Escherichia coli effector-mediated subversion of host innate immune pathways. Cell Microbiol. 2015;17(3):318–332. .PubMed PMID: 25588886 25588886 10.1111/cmi.12412

[7] Wales AD, Woodward MJ, Pearson GR Attaching-effacing bacteria in animals. PubMed PMID: 15629476 J Comp Pathol. 2005;132 1:1–26. 15629476 10.1016/j.jcpa.2004.09.005 PMC7118730

[8] Luperchio SA, Schauer DB Molecular pathogenesis of *Citrobacter rodentium* and transmissible murine colonic hyperplasia. Microbes Infect. 2001;3(4):333–340. PubMed PMID: 11334751 11334751 10.1016/s1286-4579(01)01387-9

[9] Yang J, Tauschek M, Hart E, et al Virulence regulation in *Citrobacter rodentium*: the art of timing. Microb Biotechnol. 2010;3(3):259–268. .PubMed PMID: 21255326; PubMed Central PMCID: PMCPMC3815369 21255326 10.1111/j.1751-7915.2009.00114.x PMC3815369

[10] Vallance BA, Finlay BB Exploitation of host cells by enteropathogenic Escherichia coli. Proc Natl Acad Sci U S A. 2000;97(16):8799–8806. .PubMed PMID: 10922038; PubMed Central PMCID: PMCPMC34015 10922038 10.1073/pnas.97.16.8799 PMC34015

[11] Gaytan MO, Martinez-Santos VI, Soto E, et al Type three secretion system in attaching and effacing pathogens. Front Cell Infect Microbiol. 2016;6:129 .PubMed PMID: 27818950; PubMed Central PMCID: PMCPMC5073101 27818950 10.3389/fcimb.2016.00129 PMC5073101

[12] Navarro-Garcia F, Serapio-Palacios A, Ugalde-Silva P, et al Actin cytoskeleton manipulation by effector proteins secreted by diarrheagenic Escherichia coli pathotypes. Biomed Res Int. 2013;2013:374395 .PubMed PMID: 23509714; PubMed Central PMCID: PMCPMC3591105 23509714 10.1155/2013/374395 PMC3591105

[13] Huet E, Boulay F, Barral S, et al The role of beta-arrestins in the formyl peptide receptor-like 1 internalization and signaling. Cell Signal. 2007;19(9):1939–1948. .PubMed PMID: 17594911 17594911 10.1016/j.cellsig.2007.05.006

[14] Rabiet MJ, Huet E, Boulay F Human mitochondria-derived N-formylated peptides are novel agonists equally active on FPR and FPRL1, while Listeria monocytogenes-derived peptides preferentially activate FPR. Eur J Immunol. 2005;35(8):2486–2495. .PubMed PMID: 16025565 16025565 10.1002/eji.200526338

[15] Dahlgren C, Gabl M, Holdfeldt A, et al Basic characteristics of the neutrophil receptors that recognize formylated peptides, a danger-associated molecular pattern generated by bacteria and mitochondria. Biochem Pharmacol. 2016;114:22–39. .PubMed PMID: 27131862 27131862 10.1016/j.bcp.2016.04.014

[16] Ye RD, Boulay F, Wang JM, et al International union of basic and clinical pharmacology. LXXIII. Nomenclature for the formyl peptide receptor (FPR) family. Pharmacol Rev. 2009;61(2):119–161. .PubMed PMID: 19498085; PubMed Central PMCID: PMCPMC2745437 19498085 10.1124/pr.109.001578 PMC2745437

[17] Chen K, Liu M, Liu Y, et al Formylpeptide receptor-2 contributes to colonic epithelial homeostasis, inflammation, and tumorigenesis. J Clin Invest. 2013;123(4):1694–1704. .PubMed PMID: 23454745; PubMed Central PMCID: PMCPMC3613917 23454745 10.1172/JCI65569 PMC3613917

[18] Gao JL, Lee EJ, Murphy PM Impaired antibacterial host defense in mice lacking the N-formylpeptide receptor. J Exp Med. 1999;189(4):657–662. PubMed PMID: 9989980; PubMed Central PMCID: PMCPMC2192926 9989980 10.1084/jem.189.4.657 PMC2192926

[19] Weiss E, Kretschmer D Formyl-peptide receptors in infection, inflammation, and cancer. Trends Immunol. 2018;39(10):815–829. .Epub 2018/09/10; PubMed PMID: 30195466 30195466 10.1016/j.it.2018.08.005

[20] Weiss E, Hanzelmann D, Fehlhaber B, et al Formyl-peptide receptor 2 governs leukocyte influx in local *Staphylococcus aureus* infections. Faseb J. 2018;32(1):26–36. .Epub 2017/09/01; PubMed PMID: 28855276; PubMed Central PMCID: PMCPMC5731131 28855276 10.1096/fj.201700441R PMC5731131

[21] Becker EL, Forouhar FA, Grunnet ML, et al Broad immunocytochemical localization of the formylpeptide receptor in human organs, tissues, and cells. Cell Tissue Res. 1998;292(1):129–135. PubMed PMID: 9506920 9506920 10.1007/s004410051042

[22] Rossi FW, Prevete N, Montuori N, et al Hp(2-20) peptide of *Helicobacter pylori* and the innate immune receptors: specific role(s) of the formyl peptide receptors. Infez Med. 2012;20 Suppl 2:19–25. PubMed PMID: 23042002 23042002

[23] Babbin BA, Jesaitis AJ, Ivanov AI, et al Formyl peptide receptor-1 activation enhances intestinal epithelial cell restitution through phosphatidylinositol 3-kinase-dependent activation of Rac1 and Cdc42. J Immunol. 2007;179(12):8112–8121. PubMed PMID: 18056353 18056353 10.4049/jimmunol.179.12.8112

[24] Gronert K, Gewirtz A, Madara JL, et al Identification of a human enterocyte lipoxin A4 receptor that is regulated by interleukin (IL)-13 and interferon gamma and inhibits tumor necrosis factor alpha-induced IL-8 release. J Exp Med. 1998;187(8):1285–1294. PubMed PMID: 9547339; PubMed Central PMCID: PMCPMC2212233 9547339 10.1084/jem.187.8.1285 PMC2212233

[25] Chen K, Le Y, Liu Y, et al A critical role for the g protein-coupled receptor mFPR2 in airway inflammation and immune responses. J Immunol. 2010;184(7):3331–3335. .PubMed PMID: 20200280 20200280 10.4049/jimmunol.0903022 PMC7330933

[26] Navabi N, McGuckin MA, Linden SK Gastrointestinal cell lines form polarized epithelia with an adherent mucus layer when cultured in semi-wet interfaces with mechanical stimulation. PLoS One. 2013;8(7):e68761 PubMed PMID: 23869232; PubMed Central PMCID: PMC3712011 23869232 10.1371/journal.pone.0068761 PMC3712011

[27] Quintana-Hayashi MP, Linden SK Differentiation of gastrointestinal cell lines by culture in semi-wet interface. Methods Mol Biol. 2018;1817:41–46. Epub 2018/ 07/01 PubMed PMID: 29959701. 29959701 10.1007/978-1-4939-8600-2_5

[28] Gustafsson JK, Navabi N, Rodriguez-Pineiro AM, et al Dynamic changes in mucus thickness and ion secretion during *Citrobacter rodentium* infection and clearance. PLoS One. 2013;8(12):e84430 .PubMed PMID: 24386378; PubMed Central PMCID: PMC3875541 24386378 10.1371/journal.pone.0084430 PMC3875541

[29] Maiti AK, Sharba S, Navabi N, et al IL-4 protects the mitochondria against TNFalpha and IFNgamma induced insult during clearance of infection with *Citrobacter rodentium* and Escherichia coli. Sci Rep. 2015;5:15434 .PubMed PMID: 26481427; PubMed Central PMCID: PMCPMC4613366 26481427 10.1038/srep15434 PMC4613366

[30] Bouladoux N, Harrison OJ, Belkaid Y The mouse model of infection with *Citrobacter rodentium*. Curr Protoc Immunol. 2017;119:19 5 1–5 25. .PubMed PMID: 29091261; PubMed Central PMCID: PMCPMC5667658 29091261 10.1002/cpim.34 PMC5667658

[31] Eckmann L Animal models of inflammatory bowel disease: lessons from enteric infections. Ann N Y Acad Sci. 2006;1072:28–38. .PubMed PMID: 17057188 17057188 10.1196/annals.1326.008

[32] Quintana-Hayashi MP, Navabi N, Mahu M, et al Neutrophil elastase and IL17 expressed in the pig colon during Brachyspira hyodysenteriae infection synergistically with the pathogen induce increased mucus transport speed and production via MAPK3. Infect Immun. 2017. doi:10.1128/IAI.00262-17 PubMed PMID: 28559407. PMC5520435 28559407

[33] Navabi N, Johansson ME, Raghavan S, et al *Helicobacter pylori* infection impairs the mucin production rate and turnover in the murine gastric mucosa. Infect Immun. 2013;81(3):829–837. .PubMed PMID: 23275091; PubMed Central PMCID: PMCPMC3584886 23275091 10.1128/IAI.01000-12 PMC3584886

[34] Hang HC, Yu C, Kato DL, et al A metabolic labeling approach toward proteomic analysis of mucin-type O-linked glycosylation. Proc Natl Acad Sci U S A. 2003;100(25):14846–14851. .PubMed PMID: 14657396; PubMed Central PMCID: PMCPMC299823 14657396 10.1073/pnas.2335201100 PMC299823

[35] Johansson ME Fast renewal of the distal colonic mucus layers by the surface goblet cells as measured by *in vivo* labeling of mucin glycoproteins. PloS One. 2012;7(7):e41009 .PubMed PMID: 22815896; PubMed Central PMCID: PMC3398881 22815896 10.1371/journal.pone.0041009 PMC3398881

[36] Leoni G, Alam A, Neumann PA, et al Annexin A1, formyl peptide receptor, and NOX1 orchestrate epithelial repair. J Clin Invest. 2013;123(1):443–454. .PubMed PMID: 23241962; PubMed Central PMCID: PMCPMC3533303 23241962 10.1172/JCI65831 PMC3533303

[37] Babbin BA, Laukoetter MG, Nava P, et al Annexin A1 regulates intestinal mucosal injury, inflammation, and repair. J Immunol. 2008;181(7):5035–5044. PubMed PMID: 18802107; PubMed Central PMCID: PMCPMC2778483 18802107 10.4049/jimmunol.181.7.5035 PMC2778483

[38] Schroeder BO, Birchenough GMH, Stahlman M, et al Bifidobacteria or fiber protects against diet-induced microbiota-mediated colonic mucus deterioration. Cell Host Microbe. 2018;23(1):27–40 e7. PubMed PMID: 29276171; PubMed Central PMCID: PMCPMC5764785 Epub 2017/ 12/26. . 29276171 10.1016/j.chom.2017.11.004 PMC5764785

[39] Sharba S, Navabi N, Padra M, et al Interleukin 4 induces rapid mucin transport, increases mucus thickness and quality and decreases colitis and *Citrobacter rodentium* in contact with epithelial cells. Virulence. 2019;10(1):97–117. .Epub 2019/01/23; PubMed PMID: 30665337; PubMed Central PMCID: PMCPMC6363059 30665337 10.1080/21505594.2019.1573050 PMC6363059

[40] Cattaneo F, Parisi M, Ammendola R Distinct signaling cascades elicited by different formyl peptide receptor 2 (FPR2) agonists. Int J Mol Sci. 2013;14(4):7193–7230. .PubMed PMID: 23549262; PubMed Central PMCID: PMCPMC3645683 23549262 10.3390/ijms14047193 PMC3645683

[41] Gabl M, Holdfeldt A, Sundqvist M, et al FPR2 signaling without beta-arrestin recruitment alters the functional repertoire of neutrophils. Biochem Pharmacol. 2017;145:114–122. .Epub 2017/09/01; PubMed PMID: 28855087 28855087 10.1016/j.bcp.2017.08.018

[42] Raabe CA, Groper J, Rescher U Biased perspectives on formyl peptide receptors. Biochim Biophys Acta Mol Cell Res. 2019;1866(2):305–316. Epub 2018/ 12/07 PubMed PMID: 30521870. 30521870 10.1016/j.bbamcr.2018.11.015

[43] Qin CX, May LT, Li R, et al Small-molecule-biased formyl peptide receptor agonist compound 17b protects against myocardial ischaemia-reperfusion injury in mice. Nat Commun. 2017;8:14232 .Epub 2017/02/09; PubMed PMID: 28169296; PubMed Central PMCID: PMCPMC5309721 28169296 10.1038/ncomms14232 PMC5309721

[44] Malaviya R, Ikeda T, Ross E, et al Mast cell modulation of neutrophil influx and bacterial clearance at sites of infection through TNF-alpha. Nature. 1996;381(6577):77–80. .PubMed PMID: 8609993 8609993 10.1038/381077a0

[45] Goncalves NS, Ghaem-Maghami M, Monteleone G, et al Critical role for tumor necrosis factor alpha in controlling the number of lumenal pathogenic bacteria and immunopathology in infectious colitis. Infect Immun. 2001;69(11):6651–6659. .PubMed PMID: 11598034; PubMed Central PMCID: PMCPMC100039 11598034 10.1128/IAI.69.11.6651-6659.2001 PMC100039

[46] Dann SM, Spehlmann ME, Hammond DC, et al IL-6-dependent mucosal protection prevents establishment of a microbial niche for attaching/effacing lesion-forming enteric bacterial pathogens. J Immunol. 2008;180(10):6816–6826. PubMed PMID: 18453602; PubMed Central PMCID: PMCPMC2696063 18453602 10.4049/jimmunol.180.10.6816 PMC2696063

[47] Hasnain SZ, Evans CM, Roy M, et al Muc5ac: a critical component mediating the rejection of enteric nematodes. J Exp Med. 2011;208(5):893–900. .PubMed PMID: 21502330; PubMed Central PMCID: PMCPMC3092342 21502330 10.1084/jem.20102057 PMC3092342

[48] Kim SD, Kwon S, Lee SK, et al The immune-stimulating peptide WKYMVm has therapeutic effects against ulcerative colitis. Exp Mol Med. 2013;45:e40 .PubMed PMID: 24030327; PubMed Central PMCID: PMCPMC3789265 24030327 10.1038/emm.2013.77 PMC3789265

[49] Martinez FO, Helming L, Gordon S Alternative activation of macrophages: an immunologic functional perspective. Annu Rev Immunol. 2009;27:451–483. .PubMed PMID: 19105661 19105661 10.1146/annurev.immunol.021908.132532

[50] Prescott D, McKay DM Aspirin-triggered lipoxin enhances macrophage phagocytosis of bacteria while inhibiting inflammatory cytokine production. Am J Physiol Gastrointest Liver Physiol. 2011;301(3):G487–G497. .PubMed PMID: 21659618 21659618 10.1152/ajpgi.00042.2011

[51] Maaser C, Housley MP, Iimura M, et al Clearance of *Citrobacter rodentium* requires B cells but not secretory immunoglobulin A (IgA) or IgM antibodies. Infect Immun. 2004;72(6):3315–3324. .PubMed PMID: 15155635; PubMed Central PMCID: PMCPMC415672 15155635 10.1128/IAI.72.6.3315-3324.2004 PMC415672

[52] Marasco WA, Phan SH, Krutzsch H, et al Purification and identification of formyl-methionyl-leucyl-phenylalanine as the major peptide neutrophil chemotactic factor produced by *Escherichia coli*. J Biol Chem. 1984;259(9):5430–5439. PubMed PMID: 6371005 6371005

